# Depression, stress, anxiety and their predictors in Iranian pregnant women during the outbreak of COVID-19

**DOI:** 10.1186/s40359-020-00464-8

**Published:** 2020-09-22

**Authors:** Fatemeh Effati-Daryani, Somayeh Zarei, Azam Mohammadi, Elnaz Hemmati, Sakineh Ghasemi Yngyknd, Mojgan Mirghafourvand

**Affiliations:** 1 Reproductive Health Research Center, Midwifery Department, Faculty of Nursing and Midwifery, Urmia University of Medical Sciences, Urmia, Iran; 2 Department of Midwifery, Shohada Hospital, Qom University of Medical Sciences, Qom, Iran; 3 Nursing and Midwifery Care Research Center, Midwifery and Reproductive Health Department, School of Nursing and Midwifery, Tehran University of Medical Sciences, Tehran, Iran; 4 Department of Midwifery, Faculty of Nursing and Midwifery, Tabriz University of Medical Sciences, Tabriz, Iran; 5 Population and family’s health unit, Bonab city, Bonab, Iran; 6 Social Determinants of Health Research Center, Tabriz University of Medical Sciences, Tabriz, Iran

**Keywords:** COVID-19, Pregnancy, Prevalence, Depression, Anxiety, Stress

## Abstract

**Background:**

Pregnancy as a sensitive period of a woman’s life can be affected by various psychological factors that can have adverse effects on the woman, her fetus and future baby. Since COVID-19 is a new phenomenon with limited information available, it may have adverse psychological effects on pregnant women. Therefore, this study was conducted to determine the status of depression, stress, anxiety and their predictors in Iranian pregnant women during the outbreak of COVID-19.

**Methods:**

This descriptive-analytical cross-sectional study was performed on 205 pregnant women covered by Tabriz health centers in Iran. The sampling method used was cluster sampling. The data collection tool was the socio-demographic characteristics questionnaire and the DASS-21 (Depression, Anxiety and Stress Scale-21), which were completed online by pregnant women. The general linear model was used to determine the predictive factors of depression, anxiety and stress.

**Results:**

The mean (SD) score of depression, stress, and anxiety were 3.91 (3.9), 6.22 (4.25), and 3.79 (3.39), respectively; the score range of 0 to 21. Depression, stress, and anxiety symptoms were observed in 32.7, 32.7, and 43.9% of the participants, respectively, with varying degrees from mild to very severe. Based on the adjusted general linear model, variables of education level, spouse’s job and marital life satisfaction were the predictors of depressive symptoms. Variables of spouse’s education level, spouse’s support, marital life satisfaction and the number of pregnancies were the predictive factors of anxiety symptoms and the variables of spouse’s education level, household income sufficiency, spouse’s support and marital life satisfaction were predictors of stress symptoms.

**Conclusions:**

Considering the role of marital life satisfaction, high level of spouse’s education and income in reducing symptoms of stress, anxiety, and depression in pregnant women in critical situations such as the prevalence of COVID-19, it seems that using strategies to promote marital life satisfaction and socio-economic status can play an effective role in controlling anxiety and reducing stress and depression in pregnant women.

## Background

The SARS-CoV-2 is a coronavirus belonging to the group of beta-coronaviruses. COVID-19 is the third most well-known animal coronavirus disease after SARS and MERS, both of which belong to the group of beta-coronaviruses [1]. The exact route of transmission of the disease is not yet known, but it is thought that COVID-19 can be transmitted through droplets, close contact, aerosols, and possibly through feces-mouth, and patients in the incubation period can transfer the virus to others [2, 3].

COVID-19 is rapidly advancing in the world and its mortality rate is increasing day by day. During the high prevalence of pandemics, different groups of the population, including pregnant women, as a vulnerable group, are exposed to high levels of psychological damage [4]. The unknown nature of the virus, as well as the lack of adequate information about its transmission, reproduction, risk factors, mortality, and disease-causing effects on pregnancy and the fetus [5] can pose risks not only to people’s physical health but also to their mental health. It can lead to psychological effects, including stress, anxiety and depression [6].

Although psychological change is one of the major changes in mothers during pregnancy, psychological care should not be neglected as these changes can lead to damage [7]; because stress, anxiety, and depression are likely to have adverse effects on the mother and fetus. Complications of stress during pregnancy include preterm labor, low birth weight, and delayed neuropsychiatric development in children born to these mothers [8]. Depression during pregnancy can also have adverse effects on the fetus, the most important of which are preterm labor and low birth weight [9]. Anxiety during pregnancy also increases the risk of preterm labor [10], low birth weight [11], preeclampsia and cesarean delivery [12].

In various studies, depression and anxiety have been reported to be about 10% during pregnancy [13], which is considerable depending on the conditions and living environment [14], especially in the second and third trimesters of pregnancy [15]; it was considerable in women with a history of depression, too [16]. Depressed women suffer from poor physical health and poor quality of life [17].

Unfortunately, reviewing the available studies, it is found that a research investigating the pandemic effect of COVID-19 disease during pregnancy and its psychological disorders was not available in Iran. However, a Canadian study found that pregnant women had higher levels of stress, anxiety and depression compared to the time before the COVID-19 outbreak [18]. Another study in Turkey by Durankus et al. (2020) showed that the COVID-19 pandemic could cause psychological effects in pregnant women. They showed that the levels of anxiety and depression symptoms in pregnant women increased during the COVID-19 pandemic [19]. In most cases, depression, anxiety, and stress are not detected or treated during pregnancy [7]. However, it is possible to improve the health of mothers and infants by identifying those women who have symptoms of anxiety and depression, and their risk factors during pregnancy [13].

Consequently, considering the effects of mental disorders on pregnancy and infancy, it is possible to improve the psychological state of pregnant women, prevent complications by recognizing the psychological status of pregnant women, provide information and emotional support and other psychological interventions, and help to improve the mental state of mothers, and prevent unwanted complications. Therefore, this study was conducted to determine the status of depression, stress, anxiety and their predictive factors in pregnant women during the outbreak of COVID-19 in Iran.

## Methods

### Study design and the participants

This cross-sectional study was conducted after obtaining a license from the ethics committee of the Vice Chancellor for Research and Technology of Tabriz University of Medical Sciences (code: IR.TBZMED.REC.1398.1303) on 205 pregnant women who had a file in the health centers of Tabriz-Iran in 2019. Tabriz is one of the metropolises of Iran in which about 1897 people between March to April (The last day of sampling in this study) were identified with this disease during the COVID-19 pandemic [20].

The criteria for entering the study included having a file in the health centers of Tabriz, the desire to participate in the study, having a mobile phone and a healthy pregnancy. The exclusion criteria were a history of mental illness, medical problems during pregnancy, and high-risk pregnancies.

### Sampling

Sampling method was a two-step cluster sampling. Twenty-two health centers (one-fourth of all health centers) selected randomly using the www.random.org website. The researcher extracted the list of pregnant women covered by the centers along with their phone numbers through the Integrated Health System (SIB) and randomly selected the women based on the sample size determined for each center. Then, the researcher made phone calls to the selected women and, while briefly explaining the objectives of the research and the confidentiality of the information, examined them in terms of eligibility criteria and, if eligible, they would participate in the study. Since it was not possible to do the research in the traditional way and complete the paper questionnaire, they were asked to go online to answer the socio-demographic characteristics questionnaire, and DASS-21 depression anxiety and stress questionnaire, the links of which was sent to them via WhatsApp app.

### Data collection tools

Each participant completed the socio-demographic and obstetrics characteristics questionnaire and the DASS-21 (Depression, Anxiety and Stress Scale-21) by the link to the questionnaires.

The socio-demographic characteristics questionnaire included questions on age, level of education, job, spouse’s age, level of education and job, sufficiency of monthly income for living expenses (this variable was measured by using a subjective item categorized in three levels including completely sufficient, fairy sufficient and insufficient), spouse’s support level (this variable was measured by using a subjective item categorized in four levels including extremely high, high, moderate and poor), marital life satisfaction (this variable was measured by using a subjective item categorized in four levels including extremely high, high, moderate and poor), and obstetrics questions included the number of pregnancies, gestational age, and sex of the fetus based on ultrasound.

The DASS-21 is a shortened version of DASS-42 and includes 21 questions and 3 subscales of stress, depression, and anxiety (7 questions for each subscale). The score for each question is a score from not at all (0) to very high (3). The score is calculated for each scale separately and the overall score is not calculated. The minimum score for each subscale is zero and the maximum is 21, and a higher score indicates a worse situation [21]. The DASS21 questionnaire is commonly used in the pregnant population, especially in Iran, due to the limited number of questions and simple sentences with simultaneous assessment of stress, anxiety and depression [22]. This instrument is in the public domain, and therefore, it can be freely used in research or practice [23]. This questionnaire can also be used in non-clinical populations [22, 23]. In Iran, the validity of DASS has been confirmed using forward-backward translation, factor analysis and criterion validity. The correlation between the Beck Depression Inventory (BDI) and the depression subscale was 0.7, in the range of the Zung Self-Rating Anxiety Scale (SAS), the anxiety subscale was 0.67 and in the range of the Perceived Stress Scale (PSS), and the stress subscale was 0.49. In addition, its reliability was reported to be 0.73 for the anxiety subscale, 0.81 for the depression subscale and 0.81 for the stress subscale [24]. Its reliability in pregnant women in Tabriz (the setting of this study) for the variables of depression, anxiety and stress has been calculated as 0.80, 0.72 and 0.80, respectively [25]. In this study, the internal reliability using Cronbach’s alpha was 0.80 for the anxiety subscale, 0.78 for the depression subscale and 0.77 for the stress subscale.

### Sample size

Sample size was estimated as 97 individuals with considering d (precision) = 0.1 about the mean score of stress (*m* = 7.21), standard deviation = 3.63 [26], and α = 0.05. As it was a cluster sampling, final sample size was calculated as 194 individuals with respect to the design effect of 2.0. At the end, 205 pregnant women were studied.

### Statistical analysis

Data were analyzed using SPSS (Version 24.0, SPSS Inc., Chicago, IL). Descriptive statistics including frequency, percentage, mean and standard deviation were used to describe the socio-demographic and obstetrics characteristics of the participants, depression, anxiety and stress. Normal distribution of data was examined using skewness and kurtosis. The variables of depression, anxiety and stress didnot have a normal distribution. Therefore, logarithmic transformation based on 10 (Log 10) was used for these variables with abnormal distribution. Then, the normality of log-transformed variables was checked again and all of them showed a normal distribution. General linear model test (adjusted, unadjusted) was used to determine the relationship between socio-demographic characteristics and depression, anxiety and stress.

## Results

Between the end of March 2020 and the beginning of April 2020, 205 people (with 40% participation) were examined. None of the participants had been affected by COVID-19. The mean (standard deviation) of the participants and their spouses age were 29.3 (5.5) and 34.2 (5.6) years, respectively. About half of women (55.6%) had a university degree (44.4%) and the rest had a secondary high school and diploma degrees, and about 85% were housewives. About half of the spouses of the participants (48.3%) had a university degree and the rest (51.7%) had a high school degree, and diploma, and about half of them (45.4%) had freelance jobs. About half of women (43.9%) lived in private houses and 41.5% lived in rented houses, and about two-thirds of women (62.9%) reported relatively sufficient family income. According to ultrasound, 42% of fetuses were male. More than two-thirds of participants (70.3%) were in the second half of their pregnancy, and more than half (57.1%) experienced their first pregnancy. Two-thirds reported high and very high levels of marital support (69.8) and marital life satisfaction (75.1) (Table 1).

**Table 1:** Socio-demographic characteristics of the pregnant women (*n* = 205)

Characteristic	N (%)
**Age** (Year)	
< 25	52 (25.4)
25–35	120 (58.5)
> 35	33 (16.1)
**Mean (SD)**^**a**^	29.3 (5.5)
**Education level**	
Secondary	12 (5.9)
High school	18 (8.8)
Diploma	61 (29.8)
University	114 (55.6)
**Job**	
Housewife	174 (84.9)
Employed	31 (15.1)
**Residence**	
Personal	90 (43.9)
Rental	85 (41.5)
Other^b^	30 (14.6)
**Sufficiency of income for expenses**	
Completely sufficient	24(11.7)
Fairy sufficient	129 (62.9)
Insufficient	52 (25.4)
**Husband’s age** (Year)	
< 30	116 (56.6)
30–35	56 (27.3)
> 35	33 (16.1)
**Mean (SD)**	34.2 (5.6)
**Spouse’s education level**	
Secondary	24 (11.7)
High school	27 (13.2)
Diploma	55 (26.8)
University	99 (48.3)
**Spouse’s job**	
Clerk	45 (22.0)
Worker	40 (19.5)
Shopkeeper	27 (13.2)
Other^c^	93 (4.4)
**Marital life satisfaction**	
Extremely high	72 (35.1)
High	82.0 (40.0)
Moderate	44 (21.5)
Poor	7 (3.4)
**Number of pregnancies**	
1	117 (57.1)
2	71 (34.6)
≥3	17 (8.3)
**Gestational Age** (Week)	
< 14	16 (7.8)
14–28	85 (41.5)
> 28	104 (50.7)
**Fetal sex**	
Female	78 (38.0)
Male	86 (42.0)
Unknown	41 (20.0)
**Spouse’s support**	
Extremely high	68 (3.2)
high	75 (36.6)
Moderate	54 (26.3)
poor	8 (3.9)

^a^All data indicates number (percent), unless specified

^b^Others indicate residence in parents’ house, relatives’ house and corporate house

^c^Others includes occupations such as construction, painter, agriculture, etc. (3 cases were unemployed)

The mean (standard deviation) score of depression, stress and anxiety were 3.91 (3.9), 6.22 (4.25), and 3.79 (3.39), respectively, from the score range of 0 to 21. Based on the scores obtained, they were divided into normal, mild, moderate, severe and very severe subgroups; 67.3% of women had normal status and 32.7% had symptoms of depression. In addition, 67.3% of people were normal in relation to stress and 32.7% of them had symptoms of stress. In the anxiety test, 56.1% of participants were normal and 43.9% of them had symptoms of anxiety (Table 2). The results of the present study showed no statistically significant relationship between pregnancy trimester and depression, stress and anxiety (*P* > 0.05).

**Table 2:** Different levels of depression, stress, and anxiety in pregnant women visiting Tabriz health centers during the outbreak of COVID-19 in Iran (*n*=205)

Characteristic	N (%) ^**a**^	Med (P25-P75) ^**b**^
**Depression (0-21)**	**3.91 (3.9)**^**c**^	**3 (1-6)**
Normal	138 (67.3)	
Mild	26 (12.7)	
Moderate	22 (10.7)	
Severe	15 (7.3)	
Extremely Severe	4 (2.0)	
**Stress (0-21)**	**6.22 (4.25)**^**c**^	**5 (3-9.5)**
Normal	138 (67.3)	
Mild	16 (7.8)	
Moderate	32 (15.6)	
Severe	16 (7.8)	
Extremely Severe	3 (1.5)	
**Anxiety (0-21)**	**3.79 (3.39)**^**c**^	**3 (1-6)**
Normal	115 (56.1)	
Mild	36 (17.6)	
Moderate	25 (12.2)	
Severe	13 (6.3)	
Extremely Severe	16 (7.8)	

^a^ Number (Percent)

^b^ Median (Percentiles 25- Percentiles 75)

^c^ The numbers indicate mean (standard deviation)

According to the unadjusted general linear model, there was a significant relationship between depression scores and spouse’s level of education, spouse’s job, spouse’s support, and marital life satisfaction (*P* < 0.05). Based on the adjusted general linear model, and by adjusting other variables, variables of spouse’s level of education, spouse’s job, and marital life satisfaction have been significantly associated with depression scores (*P* < 0.05) and they were able to predict 24.7% of the variance of depression score in pregnant women during the prevalence of COVID-19.

In addition, according to the unadjusted general linear model, there was a significant relationship between the anxiety score and the spouse’s level of education, spouse’s support, marital life satisfaction and the number of pregnancies (*P* < 0.05). Based on the adjusted general linear model, and by adjusting other variables, the four variables of spouse’s educational level, spouse’s support, marital life satisfaction and number of pregnancies were significantly associated with the anxiety score (*P* < 0.05) and were able to predict 19.0% of anxiety score variance in pregnant women during the prevalence of COVID-19.

Based on the unadjusted general linear model, there was a significant relationship between the stress score and age, education level, spouse’s age, spouse’s educational level, spouse’s job, household income sufficiency, spouse’s support, and marital life satisfaction (*P* < 0.05). Based on the adjusted general linear model, and by adjusting other variables, the variables of spouse’s level of education, sufficiency of household income, spouse’s support and marital life satisfaction have a significant relationship with stress score (*P* < 0.05) and it was possible to predict 21.6% of stress score variance in pregnant women during the prevalence of COVID-19 (Table 3).

**Table 3:** Predictors of stress, anxiety and depression based on general linear model in pregnant women visiting Tabriz health centers during the outbreak of COVID-19 in Iran (*n* = 205)

Characteristic	Unadjusted		Adjusted	
	β (95% CI)	***p***-value	β (95% CI)	***p***-value
**Depression**				
**Spouse’s education level (References:** University**)**				
Secondary	0.47 (−1.27 to 2.22)	0.592	−0.33 (− 1.99 to 1.34)	0.697
High school	0.44 (− 1.23 to 2.10)	0.605	− 0.85 (− 2.50 to 0.80)	0.311
Diploma	−1.46 (− 2.75 to − 0.18)	0.026	− 1.77 (− 2.95 to − 0.59)	0.003
**Spouse’s job (References:** Clerk**)**				
Worker	0.94(− 0.70 to 2.58)	0.261	0.43 (− 1.17 to 2.03)	0.596
Shopkeeper	2.21 (0.37 to 4.05)	0.019	1.82 (0.14 to 3.50)	0.034
Other^a^	−0.60 (−1.98 to 0.77)	0.387	−0.01 (−1.32 to 1.30)	0.987
**Spouse’s support (References:** Moderate**)**				
Extremely high	−3.76 (− 5.01 to − 2.50)	< 0.001	− 0.87(− 2.55 to 0.80)	0.307
High	− 1.15 (− 2.38 to 0.07)	0.065	0.76(− 0.72 to 2.24)	0.314
**Marital life satisfaction (References:** Moderately**)**				
Extremely high	−4.54 (−5.82 to − 3.26)	< 0.001	− 3.98 (− 5.77 to − 2.20)	< 0.001
High	− 2.41 (− 3.65 to − 1.16)	< 0.001	− 2.74 (− 4.32 to − 1.17)	0.001
**Anxiety**				
**Spouse’s education level (References:** University**)**				
Secondary	0.87 (− 2.39 to 0.64)	0.256	− 1.85 (− 3.35 to − 0.36)	0.015
High school	−0.74 (− 2.18 to 0.070)	0.313	− 1.72 (− 3.16 to − 0.27)	0.020
Diploma	−1.26 (− 2.38 to − 0.14)	0.027	− 1.50 (− 2.55 to − 0.45)	0.005
**Spouse’s support (References:** Moderate**)**				
Extremely high	− 1.82 (− 2.94 to − 0.70)	0.002	0.42 (− 1.05 to 1.89)	0.574
High	0.65 (− 0.44 to 1.74)	0.242	1.82 (0.49 to 3.15)	0.008
**Marital life satisfaction (References:** Moderately**)**				
Extremely high	−2.72 (− 3.89 to − 1.55)	< 0.001	− 3.73 (− 5.32 to − 2.13)	< 0.001
High	−0.97 (− 2.10 to 0.17)	0.095	− 2.39 (− 3.80 to − 0.97)	0.001
**Number of pregnancies (References:** ≥3**)**				
1	−1.84 (−3.57 to − 0.11)	0.037	− 1.97 (− 3.63 to − 0.30)	0.021
2	− 1.47 (− 3.26 to 0.33)	0.109	−1.71 (− 3.41 to 0.01)	0.050
**Stress**				
**Age (References: > 35)**				
< 25	0.84 (0.78 to 2.45)	0.309	0.75(−1.19 to 2.69)	0.447
25–35	2.75 (0.95 to 4.55)	0.003	1.47(−0.40 to 3.35)	0.123
**Education level (References:** University**)**				
Secondary	−1.63(− 4.14 to 0.88)	0.202	0.82(− 2.10 to 3.70)	0.577
High school	−0.10(− 2.20 to 1.99)	0.926	0.32(−1.96 to 2.61)	0.780
Diploma	−1.84(−3.16 to −0.53)	0.006	− 1.10(0.52 to 0.36)	0.140
**Spouse’s age (References:** > 35**)**				
< 30	−3.26(− 5.81 to −0.71)	0.012	− 1.66(− 4.44 to 1.11)	0.238
30–35	0.29(− 0.91 to 1.49)	0.936	− 0.08(− 1.49 to 1.33)	0.913
**Spouse’s education level (References:** University**)**				
Secondary	−2.02(− 3.87 to − 0.17)	0.032	− 2.02(− 4.18 to 0.14)	0.067
High school	−1.13(− 2.89 to 0.64)	0.209	− 1.44(− 3.51 to 0.62)	0.170
Diploma	− 2.62(− 3.99 to − 1.25)	< 0.001	− 2.14(− 3.58 to − 0.70)	0.004
**Sufficiency of income for expenses (References:** Insufficient**)**				
Completely sufficient	1.06(− 1.00 to 3.11)	0.312	2.24(0.22 to 4.26)	0.030
Fairy sufficient	1.44(0.07 to 2.81)	0.039	1.73(0.41 to 3.06)	0.011
**Spouse’s support (References:** Moderate**)**				
Extremely high	−2.06(− 3.47 to − 0.64)	0.005	− 0.04(− 1.96 to 1.87)	0.096
High	0.76(− 0.63 to 2.14)	0.282	1.80(0.14 to 3.45)	0.033
**Marital life satisfaction (References:** Moderately**)**				
Extremely high	−2.86(− 4.34 to −1.38)	< 0.001	−3.85(− 5.90 to − 1.79)	< 0.001
High	−0.70(− 2.15 to 0.74)	0.336	−2.51(− 4.30 to − 0.73)	0.006

^a^Others includes occupations such as construction, painter, agriculture, etc. (3 cases were unemployed)

Depression Adjusted R^2^ = 0.247

Anxiety Adjusted R^2^ = 0.190

Stress Adjusted R^2^ = 0.216

## Discussion

The results of the study showed that 32.7, 32.7, and 43.9% of the participants had depression, stress and anxiety symptoms, respectively, with varying degrees from mild to very severe. Based on the adjusted general linear model, variables of education level, spouse’s job and marital life satisfaction were the predictors of depressive symptoms. Variables of spouse’s education level, spouse’s support, marital life satisfaction and the number of pregnancies were the predictive factors of anxiety symptoms and the variables of spouse’s education level, household income sufficiency, spouse’s support and marital life satisfaction were the predictors of stress symptoms.

In the present study, 67.3% of women had normal depression and stress and 32.7% had varying degrees of depression and stress. In terms of anxiety, 56.1% of people were normal, and 43.9% suffered from varying degrees of anxiety during the pandemic of SARS-CoV-2 disease. In line with the present study, in Effati et al. [26] study on pregnant women and in a similar setting to the present study (Tabriz-Iran) (2018), more than half of women were normal in terms of depression, stress and anxiety and about 36, 32, and 27% of women experienced varying degrees of depression, stress, and anxiety symptoms, respectively. Comparing the two groups, women’s stress and depression symptoms levels were expected to be more severe during the coronavirus outbreak, while the severity of these problems was almost the same as when the coronavirus did not exist in the community. In this regard, it can be said that pregnant women, due to the importance of their fetus and its emotional attachment, may take care of themselves and follow the health advices of SARS-CoV-2 seriously. Therefore, they should have more peace of mind and confidence, followed by less stress, anxiety and depression.

During the COVID-19 pandemic, the results of Berthelot et al., [18] study showed that pregnant women had higher levels of stress, anxiety, and depression compared to the pregnant women who were examined before the pandemic, which is inconsistent with the results of our study. A possible reason for this discrepancy may be the cultural and social differences between our setting and their study. Another study by Durankus et al., (2020) [19] found that more than one-third of pregnant women had symptoms of depression and anxiety during the COVID 19 pandemic, which is almost in line with the findings of our study.

In a case-control study by Lee et al., during the outbreak of SARS, the results of anxiety in women who were pregnant during the outbreak of SARS were only slightly higher than in women who were pregnant before the outbreak of SARS and the rate of depression did not differ significantly between the two groups [4]. Perhaps the reason for not increasing or slightly increasing of the severity of anxiety, stress and depression symptoms during the outbreak of diseases such as SARS and COVID-19 is that the disease is new or not taken seriously by people in the first spread. Due to the newness of COVID-19 disease and the lack of a study in the field, it was not possible to interpret the results of the present study in pregnant women with similar conditions in other studies.

In the present study, there was a significant relationship between spouse’s level of education with depression, anxiety and stress symptoms. Women whose husbands had a non-university education were less likely to report depression, anxiety, and stress compared to those with a university degree. In a study by Salmalian et al., [27] there was a significant association between spousal education and depression before and after childbirth, so that as the level of education was lower, depression was higher. In a study on the general population [28], the level of education had a reverse statistical relationship with the three variables of depression, anxiety and stress. As the level of education increased, depression, stress and anxiety were reduced. The results of both studies are inconsistent with the results of the present study. Education can open people’s eyes and make them understand the situation, and increase their reaction to the events, especially in critical situations such as the prevalence of COVID-19. While people with non-university education may not have an idea of the bad condition and be less sensitive to the crisis of the outbreak of the disease, or may even be unaware of the dimensions of the crisis and the depth of the tragedy. While people with university education are expected to have more accurate follow-up of the deterioration of the situation from various sources such as scientific journals, cyberspace, media, etc. In addition, this increases the severity of depression, stress and anxiety symptoms and this causes a high level of depression, stress and anxiety in them and those around them.

In our study, there was a significant relationship between spouse’s job and symptoms of depression, so that women whose husbands were shopkeepers had more symptoms of depression than those whose husbands were employees. Salmalian et al. [27] reported a significant relationship between spouse’s job and pre and postpartum depression. Depression was more common in women whose husbands had lower-paying jobs, which is consistent with our results.

In the present study, there was a significant relationship between marital life satisfaction with depression, anxiety and stress scores during COVID-19 prevalence. Depression, stress and anxiety scores were lower in women who were satisfied and very satisfied with their lives compared to those who were moderately satisfied. In their study, Bakhshi et al. [29] showed that with increasing severity of depression among men and women, their marital life satisfaction decreased. Odinka et al. [30] in their study of low-risk women in the postpartum period also found a significant association between the severity of depression and anxiety and marital life satisfaction. The results of both studies were consistent with the present study.

In our study, anxiety and stress scores were significantly higher in women with high levels of support from their spouses than in those with moderate levels of support. However, one study reported high anxiety and fear of childbirth in women who had poor support from their husbands or dissatisfaction with their husbands’ support [31]. In addition, the results of a study showed that in 86% of pregnant women, the support of the husband during pregnancy has reduced their stress symptoms, and more than 90% of them have reported a sense of emotional security following the support of the husband [32]. The results of both studies are inconsistent with the results of our study. One of the possible causes of this mismatch could be that due to the depth of emotional relationships, high dependence and attachment to the spouse, the fear of losing him, his falling ill with COVID-19 disease is greater among those supported by spouses, and this can increase their stress and anxiety. It is also possible that the stress and anxiety caused by the COVID-19 pandemic in the mother will be so great that simply the support of the spouse cannot play an effective role in reducing it.

In our study, anxiety scores were significantly lower in women who experienced their first and second pregnancies than in those in the third and more pregnancies. Dunkel Schetter et al. [33] showed a high level of pregnancy anxiety in women during their first delivery. In their study of pregnant women, Rezaee et al. [34] did not report a difference in the number of parities between anxious and non-anxious women. Perhaps the reason for the lower anxiety in low parity in the COVID-19 pandemic in this study is the high relationship of mothers with low parity with health centers, which helps to obtain sufficient and accurate information and reduce their anxiety.

According to available sources, this study is the first to investigate the depression, stress and anxiety of Iranian pregnant women and their predictive factors during the prevalence of COVID-19, and the random sampling of participants is another strength of the study.

One of the limitations of this study is the cross-sectional nature of it, the relationships shown between socio-demographic variables with symptoms of stress, depression and anxiety cannot accurately reflect the causal relationship. Another limitation was that those who could have a mobile phone with internet connection could participate in this study. Although 100% of the women studied had a cell phone, only 60% had a phone with this feature. Therefore, as a limitation, this study may not be the representative of pregnant women in Iran in general.

In addition, the low level of participation was another limitation, as about half of pregnant women completed the questionnaire online. Perhaps the reason for this is the recent online method of collecting data in Iran, where all previous projects with pregnant women have been done in person.

## Conclusion

In the present study, marital life satisfaction and a high level of spousal education and income were associated with reduced symptoms of stress and anxiety in pregnant women. According to the results of the present study, low levels of stress, anxiety and depression in pregnant women during COVID-19 prevalence can be a sign of successful training in controlling negative emotions during crisis by health centers and mass media. On the other hand, given the effective role of marital life satisfaction in reducing stress and depression in pregnant women in times of crisis, such as the prevalence of coronavirus, it seems that educating spouses about techniques for strengthening the foundation of marital life can play an effective role in controlling worries and reducing stress, anxiety and depression in pregnant women.

## Data Availability

The data that support the findings of this study are not publicly available due to ongoing analyses but are available from the corresponding author, M. Mirghafourvand, upon reasonable request.

## Abbreviations

MERS: Middle East Respiratory Syndrome
DASS-21: Depression Anxiety and Stress Scale-21
SD: Standard Deviation
BDI: Beck Depression Inventory
SAS: Zung Self-Rating Anxiety Scale
PSS: Perceived Stress Scale
